# Conversion from spatial patterns of activity to sequences of neuronal activations using gate interneurons

**DOI:** 10.1186/1471-2202-14-S1-P3

**Published:** 2013-07-08

**Authors:** Eduardo Conde-Sousa, Paulo Aguiar

**Affiliations:** 1 Faculty of Sciences, University of Porto, Porto, Portugal; 2 Center for Mathematics of University of Porto, Porto, Portugal

## 

Activities such as playing the piano or moving a hand to a particular object and grab it require the execution of a finite sequence of actions. Long-term learning processes make the replication of these activities natural and reliable. The work presented here focus on the problem of using spatial neuronal activity patterns (specific constellations of neurons which become active within a short time window) to specify different sequences of activations on a population encoding learned elementary actions. Consider, for example, the case where each neuron or synchronized group of neurons in the premotor cortex triggers an elementary movement (e.g. basic movements of an arm). Different basic movements should be arranged in sequences depending if the goal is to grab an object from a table, or catch it in flight. Distinct complex actions are therefore specified by different sequences of neuronal activations in the same population [1,2], and each sequence must be recovered as a whole unit.

**Figure 1:**
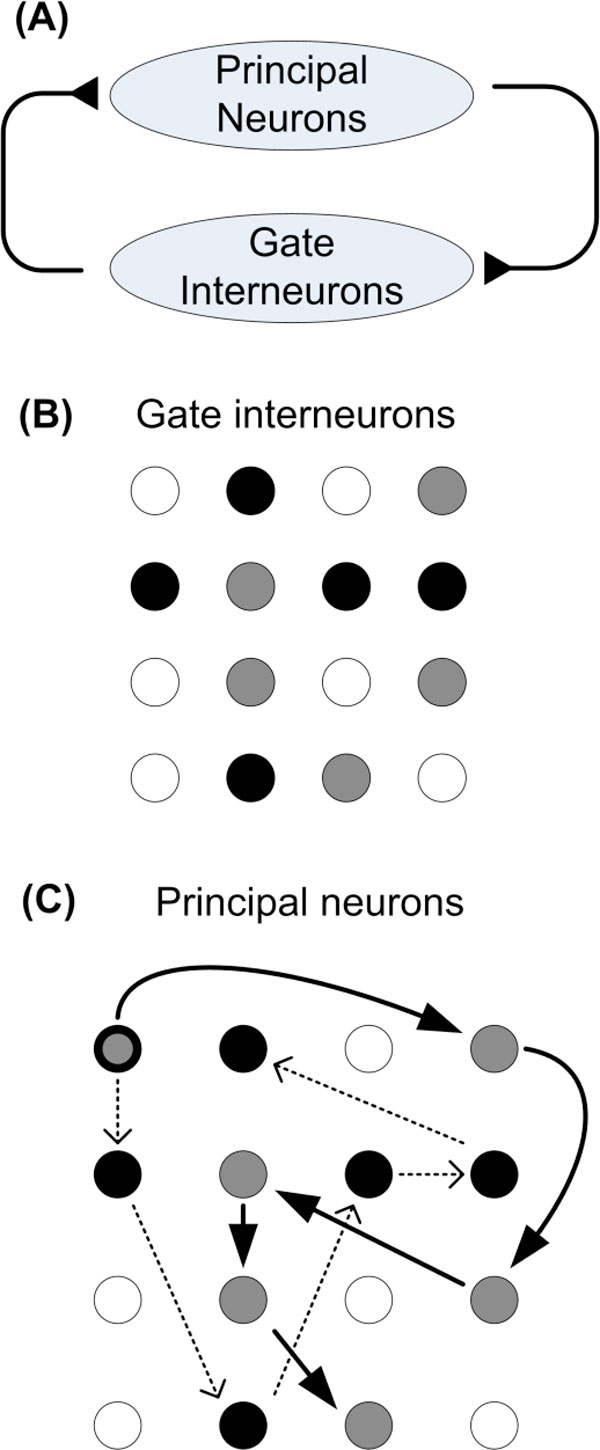
**(A) Circuit diagram**. (B), (C) Different ensembles in the gate interneuron population (gray or black spatial activations) cause different sequences of activations in principal neuron population.

Recently we proposed a novel class of excitatory interneurons, named "gate interneurons", which can play a key role in storing and recalling short-term memory sequences [3]. In the gate interneurons framework, a major pathway between principal neurons is mediated not by synapses but by the gate interneurons giving them the ability to control the flow of activations (routing) in the principal neurons population. Here we build upon this work and show that gate interneurons can be used to guide a learning process allowing the conversion of specific spatial activity patterns into specific sequences of activations. This conversion is possible because the principal neuron population establishes connections to gate interneurons which then feedback the principal neuron population (Figure 1A). Depending on which ensemble of gate interneurons is active, the feedback excitation impinges in different principal neurons, resulting in a different sequence of activations.

In the proposed model, an action that needs the activation of principal neuron A followed by the activation of principal neurons B, requires the activity of a gate interneuron responsible coding the sequential pattern AB. If gate interneuron AB is not active, then the sequence will not proceed to B and will flow to another principal neuron, depending which other gate interneurons are available. Consider, as in Figure 1C, a network of 16 principal neurons (or groups of synchronous neurons represented by one unit in the model). To execute some complex action it may be needed to follow the sequence 1→4→12→6→10→15 while in another complex action it may be required to follow the sequence 1→5→14→7→8→2, both starting in neuron 1. Depending on which gate interneurons are previously activated (spatial patterns labeled in gray and black in Figure 1B,C), the sequence may follow the first path (labeled in gray), or the second (labeled in black).
